# Crystal structure and Hirshfeld surface analysis of the coordination compound di­aqua­[5,10,15,20-tetra­kis­(4-chloro­phen­yl)porphyrinato-κ^4^*N*]magnesium(II)

**DOI:** 10.1107/S2056989026000836

**Published:** 2026-02-05

**Authors:** Mona A. Alamri

**Affiliations:** a Department of Chemistry College of Science Qassim University,Buraidah 52571 Saudi Arabia; University of Neuchâtel, Switzerland

**Keywords:** crystal structure, magnesium(II) porphyrin, di­aqua complex, inter­molecular inter­actions, Hirshfeld analysis

## Abstract

During the synthesis of the (oxalato)[5,10,15,20-tetra­kis­(4-chlor­ophen­yl)porphyrinato]magnesium(II) ([Mg(TClPP)(ox)]) complex [TClPP = 5,10,15,20-tetra­kis­(4-chloro­phen­yl)porphyrinate and ox = oxalate], the title compound, [Mg(C_44_H_24_ClMgN_4_O_2_)(H_2_O)_2_] ([Mg(TClPP)(H_2_O)_2_]), was obtained as a by-product. The di­aqua–Mg^II^ porphyrin complex crystallizes in the *I*4/*m* space group. In the asymmetric unit, except for two carbon atoms of the phenyl ring, all atoms lie on special positions. In the crystal, the [Mg(TClPP)(H_2_O)_2_] mol­ecules form layers parallel to the *a* axis. The crystal packing features C—H⋯π inter­actions involving the pyrrole rings and non-conventional O—H⋯Cl hydrogen bonds between the oxygen atom of the water axial ligands and the chloride of neighboring phenyl groups. Hirshfeld surface analysis indicates that inter­molecular contacts are dominated by H⋯H (50.2%), followed by H⋯Cl (21.6%) and H⋯C (21.2%) inter­actions, then by less chemically meaningful C⋯Cl (6.0%) contacts.

## Abstract 2

The mol­ecular structure and Hirshfeld surface analysis of the di­aqua­[5,10,15,20-tetra­kis­(4-chloro­phen­yl)porphyrinato-κ^4^*N*}magnesium(II) coordination compound with the formula [Mg(C_44_H_24_ClN_4_O_2_)(H_2_O)_2_] is described. The crystal structure of this new Mg^II^ metalloporphyrin features C—H⋯π inter­actions involving the pyrrole rings and non-conventional O—H⋯Cl hydrogen bonds between the axial aqua ligand and a nearby [Mg(TClPP)(H_2_O)_2_] mol­ecule.

## 1. Chemical context

Magnesium, the eighth most abundant element in the Earth’s crust and an essential nutrient for all living organisms, plays a central role in biological processes, most notably as the coordinating metal ion in chloro­phyll, the photosynthetic pigment that sustains life on Earth (Barker & Pilbeam, 2015 ▸). The coordination chemistry of magnesium(II), particularly within porphyrin frameworks, has thus attracted sustained scientific inter­est due to its fundamental relevance to photosynthesis and its potential in bioinspired technologies (Borah & Bhuyan, 2017 ▸).

The foundation of magnesium(II) metalloporphyrin chemistry was laid in the early to mid-20th century. One of the pioneering contributions came from Hans Fischer, whose extensive work on porphyrin synthesis and metal insertion in the 1930s and 1940s provided the first systematic routes to metalloporphyrins, although Mg^II^ complexes were often challenging to isolate due to their lability in protic media (Fischer *et al.*, 1937 ▸). Later, the structural elucidation of chloro­phyll by Robert Burns Woodward and colleagues in the 1960s, culminating in the total synthesis of chloro­phyll *a* offered profound insight into the unique coordination environment of Mg^II^ in natural porphyrinoids, notably the presence of a fifth and sixth axial ligands and the susceptibility of the Mg—N bonds to hydrolysis (Woodward *et al.*, 1960 ▸).

Unlike transition metals that form robust metalloporphyrins, Mg^II^ porphyrins are diamagnetic, *d*^0^ complexes with labile axial coordination sites, which imparts distinctive photophysical properties, but also presents synthetic challenges. Nevertheless, these attributes make Mg^II^ porphyrins particularly attractive for applications that require efficient light harvesting, energy transfer, and reversible ligand binding, all hallmarks of natural photosynthetic systems.

In recent years, synthetic Mg^II^ porphyrins have found utility beyond biology. They serve as key components in artificial photosynthetic devices, where they act as light absorbers and electron donors in photoinduced charge-separation systems (Gust *et al.*, 2001 ▸). For instance, several reported investigations have engineered tailored Mg porphyrins for integration into mol­ecular triads and tetrads that mimic the primary events of photosynthesis, achieving long-lived charge-separ­ated states relevant to solar energy conversion (Borah *et al.*, 2017 ▸). Additionally, Mg^II^ porphyrins have been employed as sensors (Gutiérrez *et al.*, 2014 ▸). More recently, they have been explored in photocatalysts for the transformation of CO_2_ to cyclic carbonates and oxazolidinones (Meher *et al.*, 2024 ▸).

Accordingly, the controlled synthesis, stabilization, and functionalization of Mg^II^ porphyrins remain active areas of research, driven by both fundamental curiosity and the pursuit of sustainable technologies inspired by nature’s design. Herein we report the synthesis, the single crystal X-ray mol­ecular structure and the Hirshfeld surfaces analysis of the title di­aqua­[{5,10,15,20-tetra­kis­(4-chloro­phen­yl)}porphyrinato-κ^4^*N*]magnesium(II) coordination compound.
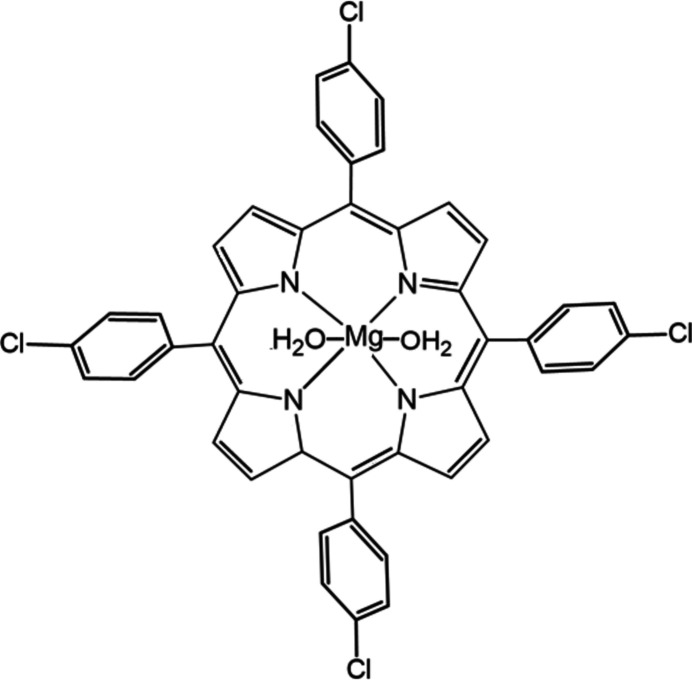


## 2. Structural commentary

**Figure 1:**
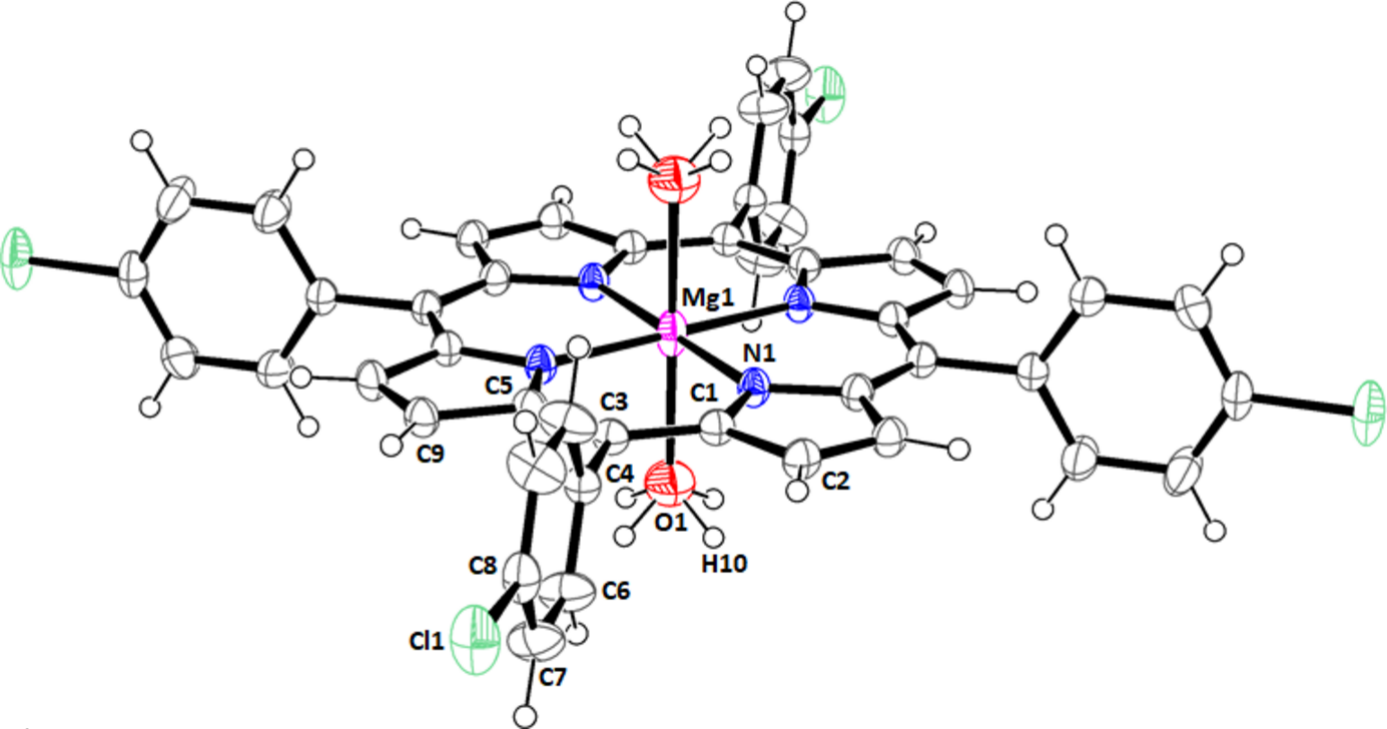
[Mg(TClPP)(H_2_O)_2_] showing the atom labelling scheme. Displacement ellipsoids are drawn at the 40% probability level. All possible positions of the disordered H atoms of the water molecules are shown.

The title compound crystallizes in the tetra­gonal space group *I4/m* (Fig. 1 ▸). The asymmetric unit comprises one quarter of the [Mg(TClPP)(H_2_O)_2_] mol­ecule leading to the formula [Mg(C_44_H_24_ClMgN_4_O_2_)(H_2_O)_2_]. The central Mg^II^ ion is coordinated to nitro­gen atoms of the porphyrin core, and to oxygen atoms of the water mol­ecules, thus showing an octa­hedral geometry.

The Mg—O(H_2_O) distance of the [Mg(TClPP)(H_2_O)_2_] complex is 2.248 (3) Å, which is in the normal range of bis­(aqua)–porphyrin complexes, *e.g.*, for the related [Mg(TBrPP)(H_2_O)_2_] (TBrP = 5,10,15,20-tetra­kis­(4-bromo­phen­yl)porphyrinate), the Mg—O(H_2_O) bond length is 2.221 (4) Å (Amiri *et al.*, 2015 ▸). Notably, the [Mg(TBrPP)(H_2_O)_2_] related species is isotypic to our TClPP–magnesium-di­aqua complex. In 2022, the structure of the [Mg(TClPP)(pyz)][Mg(TClPP)(H_2_O)_2_] (pyz = pyrazine) complex was reported, for which [Mg(TClPP)(pyz)] and [Mg(TClPP)(H_2_O)_2_] are present in the same asymmetric unit (PELVUB; Singh *et al.*, 2022 ▸). For this [Mg(TClPP)(H_2_O)_2_] coordination compound, the Mg—O(H_2_O) distance is 2.267 (5) Å, which is slightly longer than that of the title compound.

For the penta­coordinated monaqua–Mg^II^–porphyrin complex [Mg(Porph)(H_2_O)] (Porph = *meso*-aryl­porph­yr­in­ate), the Mg—O(H_2_O) bond length is shorter than those of the bis­aqua magnesium(II) porphyrins such as the [Mg(TPP)(H_2_O)_2_] complex, for which Mg—O(H_2_O) is 2.053 (5) Å (McKee & Rodley, 1988 ▸). The distance between the central Mg^2+^ ion and the N1 atom of the TClPP porphyrinate of [Mg(TClPP)(H_2_O)_2_] (Mg—N_p_) is 2.0646 (17) Å. For the related complexes [Mg(TBrPP)(H_2_O)_2_] (Amiri *et al.*, 2015 ▸) and [Mg(TClPP)(H_2_O)_2_] (Singh *et al.*, 2022 ▸), the average distances between the central Mg^2+^ ion and the four nitro­gen atoms of the pyrrole rings of the porphyrin macrocycle (Mg—Np) are 2.069 and 2.082 Å, respectively. All these Mg—Np values are typical of magnesium(II) metalloporph­yrins (Jabli *et al.*, 2022 ▸).

## 3. Supra­molecular features

**Figure 2:**
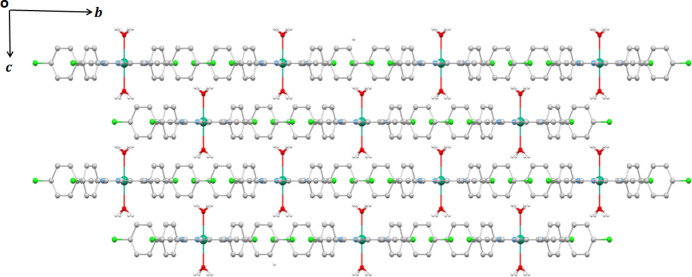
Packing viewed along the [100] direction showing the layers made by [Mg(TClPP)(H_2_O)_2_] complex mol­ecules.

**Figure 3:**
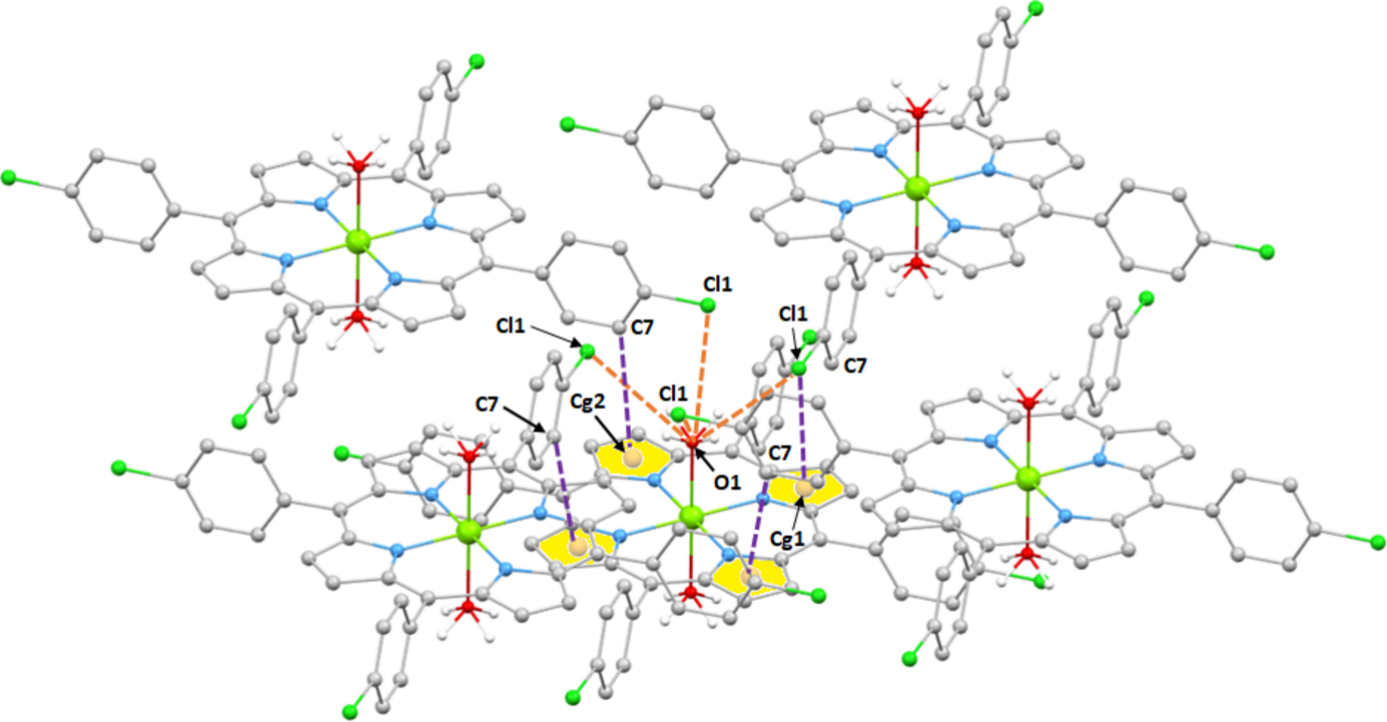
View showing the C—H⋯Cl and the C—H⋯*Cg* (*Cg* is the centroid of a pyrrol ring) inter­molecular inter­actions.

In the crystal, the [Mg(TClPP)(H_2_O)_2_] complex mol­ecules form layers parallel to the [100] direction (Fig. 2 ▸). As shown in Fig. 3 ▸, each oxygen atom of the two *trans* axial aqua ligands and the four symmetry-related atoms are involved in hydrogen bonds with the chlorine atom of a neighboring TClPP porphyrinate mol­ecule with a distance of 3.691 (2) Å (Table 1 ▸). The crystal of the new Mg^II^–di­aqua–TClPP metalloporphyrin is further consolidated by C—H⋯π inter­actions between the carbon C7 of a phenyl of a TClPP porphyrinate and the centroid of the pyrrole rings of the porphyrin core with a C7⋯centroid distance of 3.608 (2) Å (Fig. 3 ▸., Table 1 ▸).

## 4. Database survey

A survey of the Cambridge Structural Database (CSD, version 6.00, update April 2025; Groom *et al.*, 2016 ▸) revealed 11 structures of aqua magnesium(II) porphyrin complexes. Among these porphyrinic coordination complexes, four are hexa­coordinated di­aqua complexes and seven are penta­coordinated mono­aqua metalloporphyrins. The four reported di­aqua–Mg^II^–porphyrin complexes are: [Mg(T3,5-OMePP)(H_2_O)_2_] (T3,5-OMePP = 5,10,15,20-tetra­kis­(3,5-di­meth­oxy­phen­yl)porphyrinate) (GOJGEV; Borah *et al.*, 2024 ▸), [Mg(TBPP)(H_2_O)_2_] (TPBPP = 5,10,15,20-tetra­kis­(4-(benzo­yl­oxy)phen­yl)porphyrinate) (CUCZAD; Amiri *et al.*, 2015 ▸), [Mg(TPP)(H_2_O)_2_](18-C-6) (TPP = 5,10,15,20-tetra­phenyl­porphyrinate and 18-C-6 = 18-crown-6) (LERTAF; Ezzayani *et al.*, 2013 ▸) and [MgTClPP(pyz)_2_][MgTClPP(H_2_O)_2_] (pyz = pyrazine) (PELVUB; Singh *et al.*, 2022 ▸). In this latter example, one half [Mg(TClPP)(pyz)_2_] mol­ecule and one half [Mg(TClPP)(H_2_O)_2_] mol­ecule are both present in the asymmetric unit. The seven reported mono­aqua Mg^II^ metalloporphyrins are: [Mg(TPP)(H_2_O)]·2(C_6_H_7_N) (C_6_H_7_N = picoline) (DUJKUO; Ong *et al.*, 1986 ▸), [Mg(TPP)(H_2_O)]·C_3_H_6_O (GEPBUY; McKee & Rodley, 1988 ▸), [Mg(T3,5-OMePP)(H_2_O)] (GUHXAL; Borah *et al.*, 2017 ▸), [Mg(TPBP)(H_2_O)] (TPBP = 5,10,15,20-tetra­kis­(4-(benzo­yloxy)phen­yl)porphyrinate (HALDOR; Amiri *et al.*, 2022 ▸), [Mg(TMPP)(H_2_O)] (TMPP = 5,10,15,20-tetra­kis­(4-meth­oxy­phen­yl)porphyrinate) (JONKAY; Yang *et al.*, 1991 ▸), [Mg(TPP)(H_2_O)] (MGPPOR; Timkovich *et al.*, 1969 ▸), and [Mg(TTP)(H_2_O)] (TTP = 5,10,15,20-tetra­kis­(4-methyl­phen­yl)porphyrinate) (YONYAF; Meher *et al.*, 2024 ▸).

## 5. Hirshfeld surface analysis

**Figure 4:**
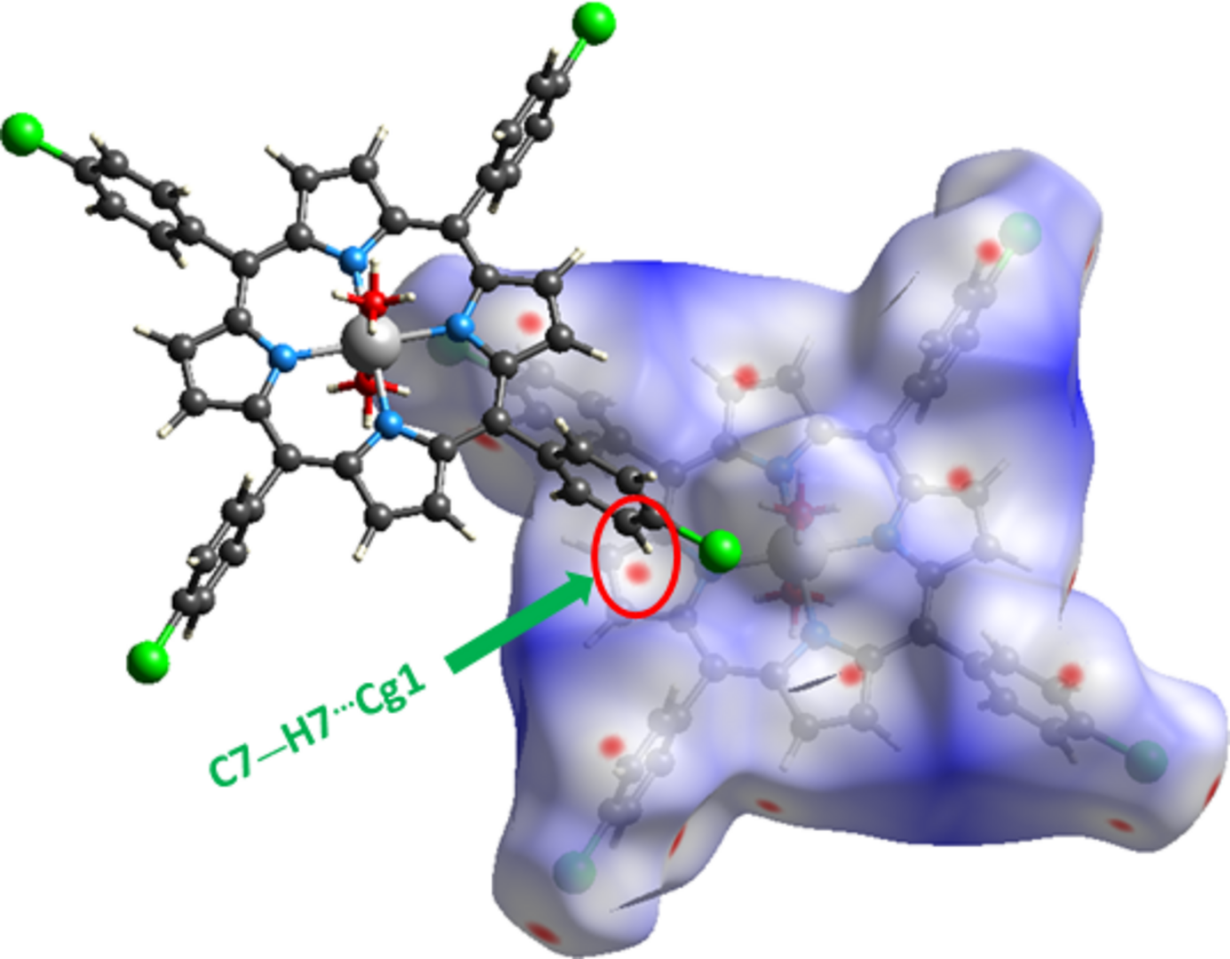
Hirshfeld surface plotted over *d*_norm_ for the title compound.

**Figure 5:**
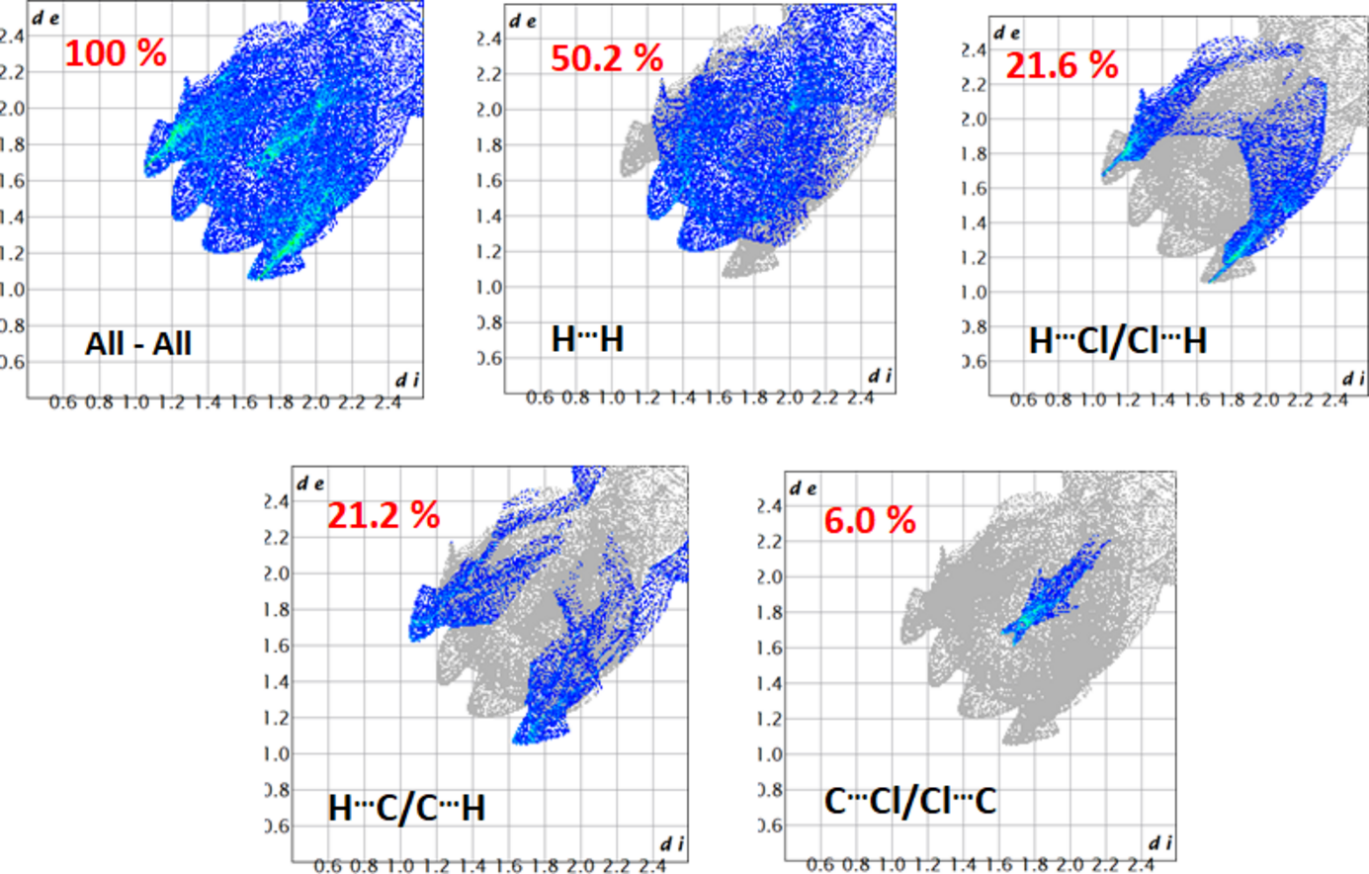
Two-dimensional fingerprint plots showing the distribution of inter­molecular inter­actions responsible for the cohesion of the title complex.

The inter­molecular inter­actions responsible for the crystal cohesion of [Mg(TClPP)(H_2_O)_2_] were also investigated using Hirshfeld surface analysis and two-dimensional fingerprint plots (Turner *et al.*, 2017 ▸). The Hirshfeld surfaces were obtained using a standard high surface resolution, mapped over *d*_norm_ (Fig. 4 ▸). As shown in Fig. 4 ▸, the red spots correspond to the non-conventional O—H⋯Cl inter­actions between the water oxygen atom and the chlorine atoms in the *para*-positions of the four TClPP phenyl rings of neighboring [Mg(TClPP)(H_2_O)_2_] mol­ecules. Similarly, the C7—H7⋯π inter­actions (Table 1 ▸) are represented as red dots. The *d*_i_*versus d*_e_ plots shown in Fig. 5 ▸ illustrate the distribution of individual inter­molecular inter­actions on the basis of fingerprint maps. The crystal structure is dominated by H⋯H (50.2%) inter­actions, followed by H⋯Cl/Cl⋯H (21.6%), H⋯C/C⋯H (21.2%) and C⋯Cl/Cl⋯C (6.0) contacts.

## 6. Synthesis and crystallization of the title complex

In order to prepare the [Mg(TClPP)(ox)] complex (ox = oxalato C_2_O_4_^2−^), a solution of [Mg(TClPP)] (100 mg, 0.128 mmol) in di­chloro­methane (40 mL) was added an excess of 18-crown-6 ether (250 mg, 0.946 mmol) and a large excess of K_2_C_2_O_4_·H_2_O (potassium oxalate monohydrate) (30 mg, 0.163 mol). The reaction mixture was stirred at room temperature for three h and at the end of the reaction, the color of the solution gradually changed from purple to blue. The resulting material was crystallized by diffusion of *n*-hexane through the di­chloro­methane solution. Single-crystal X-ray diffraction revealed that the crystals obtained correspond to the di­aqua–magnesium(II)-TClPP coordination compound. Elemental analysis calculated (%) for C_44_H_28_ClMgN_4_O_2_ (*M*_W_ = 810.83), C 65.18, H 3.48, N 6.91; found: C 65.49, H 3.61, N 7.12.

## 7. Refinement

Crystal data, data collection and structure refinement details are given in Table 2 ▸. The H-atom position of the axially bonded aqua ligand was found in difference maps and then refined with *U*_iso_(H) = 1.5*U*_eq_(O). The mol­ecular symmetry of the water mol­ecule is not compatible with the fourfold axis; hence, the occupancy of this H atom was fixed to 0.5. The H atoms attached to C atoms were fixed geometrically and treated as riding with C—H = 0.95 Å and *U*_iso_(H) = 1.5*U*_eq_(C).

## Supplementary Material

Crystal structure: contains datablock(s) I. DOI: 10.1107/S2056989026000836/tx2107sup1.cif

Structure factors: contains datablock(s) I. DOI: 10.1107/S2056989026000836/tx2107Isup3.hkl

CCDC reference: 2526366

Additional supporting information:  crystallographic information; 3D view; checkCIF report

Additional supporting information:  crystallographic information; 3D view; checkCIF report

## Figures and Tables

**Table 1:** Hydrogen-bond geometry (Å, °) *Cg*1 and *Cg*2 are the centroids of the N1,C1,C2,C9′′,C5′′ and C5,C9,C2′,C1′,N1′ rings, respectively. Symmetry codes: (′) −*y*, −1 + *x*, *z*; (′′) 1 + *y*, −*x*, *z*.

*D*—H⋯*A*	*D*—H	H⋯*A*	*D*⋯*A*	*D*—H⋯*A*
C7—H7⋯*Cg*1^i^	0.95	2.75	3.608 (2)	150
C7—H7⋯*Cg*2^ii^	0.95	2.75	3.608 (2)	150
O1—H10⋯Cl1^iii^	1.01	2.92	3.691 (2)	128

Symmetry codes: (i) 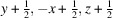; (ii) 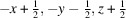; (iii) 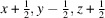.

**Table 2:** Experimental details

Crystal data	
Chemical formula	[Mg(C_44_H_24_Cl_4_N_4_)(H_2_O)_2_]
*M*_r_	810.81
Crystal system, space group	Tetragonal, *I*4/*m*
Temperature (K)	200
*a*, *c* (Å)	14.605 (2), 9.4397 (19)
*V* (Å^3^)	2013.5 (7)
*Z*	2
Radiation type	Mo *K*α
μ (mm^−1^)	0.35
Crystal size (mm)	0.30 × 0.30 × 0.30

Data collection	
Diffractometer	Bruker AXS Enraf–Nonius Kappa APEXII
Absorption correction	Multi-scan (*SADABS*; Krause *et al.*, 2015 ▸)
*T*_min_, *T*_max_	0.711, 1.000
No. of measured, independent and observed [*I* > 2σ(*I*)] reflections	8146, 1216, 1078
*R*_int_	0.032
(sin θ/λ)_max_ (Å^−1^)	0.650

Refinement	
*R*[*F*^2^ > 2σ(*F*^2^)], *wR*(*F*^2^), *S*	0.041, 0.115, 1.07
No. of reflections	1216
No. of parameters	78
No. of restraints	1
H-atom treatment	H-atom parameters constrained
Δρ_max_, Δρ_min_ (e Å^−3^)	0.32, −0.41

Computer programs: *APEX2* and *SAINT* (Bruker, 2014 ▸), *SIR2004* (Burla *et al.*, 2005 ▸) and *SHELXL2015* (Sheldrick, 2015 ▸).

## supplementary crystallographic information

### Diaqua[5,10,15,20-tetrakis(4-chlorophenyl)porphyrinato-κ4N]magnesium(II) Crystal data

**Table:**

[Mg(C_44_H_24_Cl_4_N_4_)(H_2_O)_2_]	*D*_x_ = 1.337 Mg m^−^^3^
*M**_r_* = 810.81	Mo *K*α radiation, λ = 0.71073 Å
Tetragonal, *I*4/*m*	Cell parameters from 8167 reflections
*a* = 14.605 (2) Å	θ = 2.6–27.5°
*c* = 9.4397 (19) Å	µ = 0.35 mm^−^^1^
*V* = 2013.5 (7) Å^3^	*T* = 200 K
*Z* = 2	Block, blue
*F*(000) = 832	0.30 × 0.30 × 0.30 mm

### Diaqua[5,10,15,20-tetrakis(4-chlorophenyl)porphyrinato-κ4N]magnesium(II) Data collection

**Table:**

Bruker AXS Enraf–Nonius Kappa APEXII diffractometer	1078 reflections with *I* > 2σ(*I*)
Radiation source: Incoatec ISG250	*R*_int_ = 0.032
φ and ω scans	θ_max_ = 27.5°, θ_min_ = 2.6°
Absorption correction: multi-scan (SADABS; Krause *et al.*, 2015)	*h* = −18→18
*T*_min_ = 0.711, *T*_max_ = 1	*k* = −17→18
8146 measured reflections	*l* = −9→12
1216 independent reflections	

### Diaqua[5,10,15,20-tetrakis(4-chlorophenyl)porphyrinato-κ4N]magnesium(II) Refinement

**Table:**

Refinement on *F*^2^	1 restraint
Least-squares matrix: full	Hydrogen site location: mixed
*R*[*F*^2^ > 2σ(*F*^2^)] = 0.041	H-atom parameters constrained
*wR*(*F*^2^) = 0.115	*w* = 1/[σ^2^(*F*_o_^2^) + (0.056*P*)^2^ + 1.969*P*] where *P* = (*F*_o_^2^ + 2*F*_c_^2^)/3
*S* = 1.07	(Δ/σ)_max_ < 0.001
1216 reflections	Δρ_max_ = 0.32 e Å^−^^3^
78 parameters	Δρ_min_ = −0.41 e Å^−^^3^

### Diaqua[5,10,15,20-tetrakis(4-chlorophenyl)porphyrinato-κ4N]magnesium(II) Special details

**Table:**

Geometry. All esds (except the esd in the dihedral angle between two l.s. planes) are estimated using the full covariance matrix. The cell esds are taken into account individually in the estimation of esds in distances, angles and torsion angles; correlations between esds in cell parameters are only used when they are defined by crystal symmetry. An approximate (isotropic) treatment of cell esds is used for estimating esds involving l.s. planes.

### Diaqua[5,10,15,20-tetrakis(4-chlorophenyl)porphyrinato-κ4N]magnesium(II) Fractional atomic coordinates and isotropic or equivalent isotropic displacement parameters (Å^2^)

**Table:**

	*x*	*y*	*z*	*U*_iso_*/*U*_eq_	Occ. (<1)
Mg1	0.5000	−0.5000	0.0000	0.0360 (4)	
N1	0.53951 (11)	−0.36427 (11)	0.0000	0.0283 (4)	
C4	0.33650 (13)	−0.20277 (14)	0.0000	0.0290 (4)	
C1	0.48278 (14)	−0.28960 (13)	0.0000	0.0278 (4)	
C5	0.33189 (14)	−0.37233 (14)	0.0000	0.0286 (4)	
C3	0.38633 (13)	−0.29286 (13)	0.0000	0.0275 (4)	
C8	0.24114 (15)	−0.03956 (14)	0.0000	0.0370 (5)	
C6	0.31214 (13)	−0.16133 (12)	0.1253 (2)	0.0463 (5)	
H6	0.3285	−0.1893	0.2126	0.056*	
O1	0.5000	−0.5000	0.2381 (3)	0.0583 (7)	
H10	0.5516	−0.5286	0.2938	0.070*	0.5
C7	0.26386 (14)	−0.07894 (12)	0.1262 (2)	0.0503 (5)	
H7	0.2471	−0.0508	0.2131	0.060*	
Cl1	0.17689 (4)	0.06145 (4)	0.0000	0.0588 (3)	
C2	0.53683 (15)	−0.20673 (14)	0.0000	0.0320 (5)	
H2	0.5142	−0.1457	0.0000	0.038*	
C9	0.23260 (14)	−0.37383 (14)	0.0000	0.0311 (4)	
H9	0.1931	−0.3221	0.0000	0.037*	

### Diaqua[5,10,15,20-tetrakis(4-chlorophenyl)porphyrinato-κ4N]magnesium(II) Atomic displacement parameters (Å^2^)

**Table:**

	*U*^11^	*U*^22^	*U*^33^	*U*^12^	*U*^13^	*U*^23^
Mg1	0.0233 (4)	0.0233 (4)	0.0615 (10)	0.000	0.000	0.000
N1	0.0240 (8)	0.0228 (8)	0.0381 (10)	−0.0006 (6)	0.000	0.000
C4	0.0270 (9)	0.0245 (9)	0.0354 (11)	0.0016 (7)	0.000	0.000
C1	0.0295 (10)	0.0232 (9)	0.0307 (10)	0.0003 (7)	0.000	0.000
C5	0.0262 (9)	0.0271 (9)	0.0326 (10)	0.0032 (7)	0.000	0.000
C3	0.0277 (9)	0.0246 (9)	0.0303 (10)	0.0036 (7)	0.000	0.000
C8	0.0268 (10)	0.0232 (9)	0.0609 (15)	0.0026 (8)	0.000	0.000
C6	0.0605 (11)	0.0423 (9)	0.0361 (9)	0.0202 (8)	−0.0061 (8)	−0.0041 (7)
O1	0.0619 (10)	0.0619 (10)	0.0510 (16)	0.000	0.000	0.000
C7	0.0599 (11)	0.0429 (9)	0.0481 (11)	0.0190 (8)	−0.0033 (9)	−0.0148 (8)
Cl1	0.0414 (4)	0.0271 (3)	0.1078 (7)	0.0095 (2)	0.000	0.000
C2	0.0349 (10)	0.0218 (9)	0.0393 (11)	−0.0021 (8)	0.000	0.000
C9	0.0246 (9)	0.0310 (10)	0.0377 (11)	0.0050 (8)	0.000	0.000

### Diaqua[5,10,15,20-tetrakis(4-chlorophenyl)porphyrinato-κ4N]magnesium(II) Geometric parameters (Å, º)

**Table:**

Mg1—N1^i^	2.0646 (17)	C5—C3	1.407 (3)
Mg1—N1^ii^	2.0646 (17)	C5—C9	1.450 (3)
Mg1—N1^iii^	2.0646 (17)	C8—C7	1.364 (2)
Mg1—N1	2.0646 (17)	C8—C7^iv^	1.364 (2)
Mg1—O1^i^	2.248 (3)	C8—Cl1	1.748 (2)
Mg1—O1	2.248 (3)	C6—C7	1.395 (2)
N1—C1	1.370 (3)	C6—H6	0.9500
N1—C5^ii^	1.372 (3)	O1—H10	1.0100
C4—C6^iv^	1.376 (2)	C7—H7	0.9500
C4—C6	1.376 (2)	C2—C9^ii^	1.358 (3)
C4—C3	1.504 (3)	C2—H2	0.9500
C1—C3	1.409 (3)	C9—C2^iii^	1.358 (3)
C1—C2	1.445 (3)	C9—H9	0.9500
C5—N1^iii^	1.372 (3)		
			
N1^i^—Mg1—N1^ii^	90.0	C3—C1—C2	125.05 (19)
N1^i^—Mg1—N1^iii^	90.0	N1^iii^—C5—C3	125.41 (18)
N1^ii^—Mg1—N1^iii^	180.00 (9)	N1^iii^—C5—C9	109.30 (17)
N1^i^—Mg1—N1	180.0	C3—C5—C9	125.28 (18)
N1^ii^—Mg1—N1	89.999 (1)	C5—C3—C1	126.35 (18)
N1^iii^—Mg1—N1	90.001 (1)	C5—C3—C4	116.63 (17)
N1^i^—Mg1—O1^i^	90.0	C1—C3—C4	117.01 (17)
N1^ii^—Mg1—O1^i^	90.0	C7—C8—C7^iv^	121.7 (2)
N1^iii^—Mg1—O1^i^	90.0	C7—C8—Cl1	119.11 (10)
N1—Mg1—O1^i^	90.0	C7^iv^—C8—Cl1	119.11 (10)
N1^i^—Mg1—O1	90.0	C4—C6—C7	121.01 (17)
N1^ii^—Mg1—O1	90.0	C4—C6—H6	119.5
N1^iii^—Mg1—O1	90.0	C7—C6—H6	119.5
N1—Mg1—O1	90.0	Mg1—O1—H10	121.4
O1^i^—Mg1—O1	180.0	C8—C7—C6	118.81 (17)
C1—N1—C5^ii^	107.05 (16)	C8—C7—H7	120.6
C1—N1—Mg1	126.55 (13)	C6—C7—H7	120.6
C5^ii^—N1—Mg1	126.40 (14)	C9^ii^—C2—C1	106.96 (18)
C6^iv^—C4—C6	118.6 (2)	C9^ii^—C2—H2	126.5
C6^iv^—C4—C3	120.67 (10)	C1—C2—H2	126.5
C6—C4—C3	120.67 (10)	C2^iii^—C9—C5	107.02 (18)
N1—C1—C3	125.28 (18)	C2^iii^—C9—H9	126.5
N1—C1—C2	109.67 (18)	C5—C9—H9	126.5

Symmetry codes: (i) −*x*+1, −*y*−1, −*z*; (ii) *y*+1, −*x*, −*z*; (iii) −*y*, *x*−1, *z*; (iv) *x*, *y*, −*z*.

### Diaqua[5,10,15,20-tetrakis(4-chlorophenyl)porphyrinato-κ4N]magnesium(II) Hydrogen-bond geometry (Å, º)

*Cg*1 and *Cg*2 are the centroids of the N1,C1,C2,C9'',C5'' and C5,C9,C2',C1',N1' rings, respectively. Symmetry codes: (') -*y*, -1 + *x*, *z*; ('') 1 + *y*, -*x*, *z*.

**Table:**

*D*—H···*A*	*D*—H	H···*A*	*D*···*A*	*D*—H···*A*
C7—H7···*Cg*1^v^	0.95	2.75	3.608 (2)	150
C7—H7···*Cg*2^vi^	0.95	2.75	3.608 (2)	150
O1—H10···Cl1^vii^	1.01	2.92	3.691 (2)	128

Symmetry codes: (v) *y*+1/2, −*x*+1/2, *z*+1/2; (vi) −*x*+1/2, −*y*−1/2, *z*+1/2; (vii) *x*+1/2, *y*−1/2, *z*+1/2.
